# Reversal of left ventricular hypertrophy with sacubitril/valsartan vs. standard antihypertensive agents in hypertension: a systematic review and network meta-analysis

**DOI:** 10.3389/fcvm.2026.1794041

**Published:** 2026-04-14

**Authors:** Weizhi Tang, Weibin Qin, Yun Su, Feifei Yang, Guixin He

**Affiliations:** 1 Graduate School, Guangxi University of Chinese Medicine, Nanning, China; 2 Department of Cardiovascular Medicine, The First Affiliated Hospital of Guangxi University of Chinese Medicine, Nanning, China; 3 Department of Cardiovascular Medicine, Guangxi International Zhuang Medical Hospital, Nanning, China

**Keywords:** cardiovascular remodeling, hypertension, left ventricular hypertrophy, network meta-analysis, sacubitril/valsartan

## Abstract

**Objective:**

To systematically evaluate the relative efficacy and impact on cardiac function of sacubitril/valsartan (Sac/Val) vs. active antihypertensive comparators represented in the eligible evidence base for reversing left ventricular hypertrophy (LVH) in patients with hypertension using network meta-analysis.

**Methods:**

PubMed, Embase, The Cochrane Library, CNKI, Wanfang Data, and SinoMed databases were searched from inception to December 2025 for randomized controlled trials (RCTs) evaluating sacubitril/valsartan vs. active antihypertensive comparators in patients with essential hypertension and cardiovascular remodeling. The primary outcome was the change in left ventricular mass index (LVMI). Network meta-analysis was performed using STATA 18.0 software based on the frequentist framework. Given the clinical heterogeneity in imaging assessment modalities, a random-effects model was employed to calculate the weighted mean difference (MD) and 95% confidence intervals (CI). The surface under the cumulative ranking curve (SUCRA) was used as a supportive ranking metric, whereas comparative interpretation primarily relied on effect estimates and their confidence intervals.

**Results:**

Eleven RCTs involving 851 patients were included. The network meta-analysis showed that Sac/Val achieved greater LVMI regression than Amlodipine (MD = −22.54 g/m^2^, 95% CI: −40.23, −4.86) and Valsartan (MD = −11.34 g/m^2^, 95% CI: −21.45, −1.23) in reversing LVMI. Compared with Enalapril and Olmesartan, Sac/Val also showed numerically greater LVMI regression, but these differences were not statistically significant. Sac/Val had the highest SUCRA value (96.4%); however, rankings were interpreted descriptively only, while comparative interpretation was primarily based on effect sizes and confidence intervals. Secondary outcome analysis indicated that while Sac/Val effectively reduced systolic and diastolic blood pressure, it had no significant impact on left ventricular ejection fraction (LVEF) (*P* > 0.05).

**Conclusion:**

In hypertensive patients with cardiovascular remodeling, sacubitril/valsartan was associated with greater LVMI regression than amlodipine and valsartan within the current network, whereas comparisons with enalapril and olmesartan remained inconclusive. Given the substantial heterogeneity, evidence of network incoherence, and low-to-very-low certainty of the main comparisons, these results should be regarded as tentative rather than definitive.

**Systematic Review Registration:**

PROSPERO CRD420261281426.

## 1 Introduction

Left ventricular hypertrophy (LVH) induced by chronic pressure overload represents a pivotal determinant of cardiovascular events in patients with hypertension. Epidemiological data indicate that the presence of LVH is associated with a more than two-fold increase in cardiovascular risk—a risk that correlates linearly with elevated left ventricular mass index (LVMI) (1, 2). Consequently, the ultimate goal of antihypertensive therapy extends beyond mere blood pressure reduction to encompass the dual restoration of cardiac structure and function (3).

While first-line pharmacological agents—including angiotensin-converting enzyme inhibitors (ACEIs), angiotensin receptor blockers (ARBs), and calcium channel blockers (CCBs)—demonstrate established efficacy in blood pressure control (4, 5), their capacity to reverse LVH and attenuate myocardial fibrosis varies significantly. Interventions targeting a single mechanism often fail to completely arrest the remodeling process, resulting in residual structural impairment in many patients even after achieving blood pressure targets (6). This clinical reality has necessitated the active exploration of therapeutic strategies with more comprehensive mechanistic profiles.

Sacubitril/valsartan (Sac/Val), the first-in-class angiotensin receptor-neprilysin inhibitor (ARNI), has demonstrated superior anti-remodeling capabilities in the management of heart failure, attributed to its dual mechanism of neprilysin inhibition and renin-angiotensin-aldosterone system (RAAS) blockade (7). Specifically in hypertensive populations, the REVERSE-LVH trial suggested that Sac/Val may improve LVMI and myocardial tissue remodeling compared with conventional monotherapy, although the extent to which these effects are independent of blood pressure reduction remains uncertain (8).

Although several randomized controlled trials (RCTs) have evaluated individual antihypertensive agents, comparative evidence across active treatments for regression of hypertensive cardiac remodeling remains limited. Accordingly, clinicians lack sufficiently robust comparative evidence to inform treatment selection. Using a network meta-analysis approach, this study aimed to compare sacubitril/valsartan with active antihypertensive comparators represented in the currently available randomized evidence base in terms of LVMI regression. The objective was to provide a comparative synthesis to inform, rather than determine, treatment selection in clinical practice.

## 2 Materials and methods

### 2.1 Study design and registration

This systematic review and network meta-analysis (NMA) is reported in strict adherence to the Preferred Reporting Items for Systematic Reviews and Meta-Analyses (PRISMA) extension statement for network meta-analyses (9). The study protocol was prospectively registered in the PROSPERO database (No. CRD420261281426).

### 2.2 Search strategy

The search strategy was formulated based on the PICOS principle. Two investigators independently searched PubMed, Embase, The Cochrane Library, CNKI, Wanfang Data, and SinoMed databases, covering the period from inception to December 2025. We employed a combination of Medical Subject Headings (MeSH) and free-text terms. The core English search terms included “Sacubitril/Valsartan”, “Angiotensin Receptor Neprilysin Inhibitor”, “Left Ventricular Hypertrophy”, “Cardiovascular Remodeling”, and the names of active comparator drugs. Additionally, we identified potential grey literature by examining the reference lists of included studies and querying clinical trial registries. The search strategy was designed to be sensitive in order to identify all potentially eligible active-comparator randomized controlled trials. However, only interventions with extractable LVMI change data and sufficient comparative evidence to contribute to a connected and analyzable network were retained in the final NMA. Studies identified during screening but not represented in the final network were excluded at the full-text stage because they lacked eligible comparative data, did not report extractable outcome statistics, or could not be incorporated into a connected network structure.

### 2.3 Inclusion and exclusion

**Inclusion criteria were as follows: (1) Study Design:** Published RCTs evaluating sacubitril/valsartan or any active antihypertensive comparator in adults with essential hypertension and cardiovascular remodeling were eligible. Treatments were not restricted *a priori* to specific drug classes. After screening, only sacubitril/valsartan, valsartan, enalapril, olmesartan, and amlodipine were represented by extractable LVMI change data within a connected network and were therefore included in the final NMA. **(2) Population:** Adult patients with a confirmed diagnosis of essential hypertension and baseline evidence of cardiovascular remodeling. Remodeling was defined as meeting either of the following criteria: (1) Structural remodeling: LVH confirmed by echocardiography or cardiac magnetic resonance (CMR); (2) Functional/Vascular remodeling: Evidence of significantly increased hemodynamic load, such as pulse pressure >50 mmHg or abnormal arterial stiffness indices. Because remodeling phenotypes were not fully uniform across trials, sensitivity analyses were planned restricting the network to imaging-confirmed LVH where data permitted. **(3) Intervention and Follow-up:** Drug treatment duration of at least 12 weeks to ensure sufficient time for observing morphological changes in cardiac structure. **(4) Outcome:** The change in LVMI from baseline.

**Exclusion Criteria:** (1) Patients with heart failure with reduced ejection fraction (HFrEF) or secondary hypertension, to exclude remodeling differences driven by primary disease pathology. (2) Single-arm studies or studies in which the SD of change scores could neither be directly extracted nor derived from the reported summary statistics.

### 2.4 Data extraction and quality assessment

Literature screening and data extraction were performed independently by two investigators, with discrepancies resolved through consensus or consultation with a third investigator. Extracted data included: first author, year of publication, sample size, follow-up duration, baseline characteristics, and imaging modality. Outcomes of interest included the change in LVMI, systolic blood pressure (SBP), diastolic blood pressure (DBP), and left ventricular ejection fraction (LVEF). For continuous outcomes, the mean change from baseline to follow-up and its standard deviation were extracted whenever directly reported. When change-score SDs were unavailable, they were derived from baseline and follow-up SDs using standard methods recommended in the Cochrane Handbook. The risk of bias for included studies was assessed using the Cochrane Risk of Bias 2.0 (RoB 2.0) tool (10). Potential effect modifiers relevant to transitivity were extracted, including baseline LVMI, imaging modality, age, diabetes status or prevalence, baseline systolic and diastolic blood pressure, follow-up duration, phenotype definition, and relevant background treatment features when reported; these data are summarized comparatively in Supplementary Table S1.

### 2.5 Statistical analysis

All statistical analyses were conducted within a frequentist framework using STATA 18.0 software. For continuous variables such as LVMI, blood pressure, and ejection fraction, mean difference (MD) and 95% confidence intervals (CIs) were used as effect estimates. MD was retained because all included studies reported LVMI/LVM index on a common clinical scale (g/m²), preserving interpretability. To address potential heterogeneity related to imaging modality rather than scale, we performed modality- and phenotype-restricted sensitivity analyses instead of converting the primary outcome to SMD. Given the anticipated clinical heterogeneity inherent in imaging modalities (echocardiography vs. CMR) and baseline patient characteristics, a DerSimonian-Laird random-effects model was used for the primary analyses. When the SD of change scores was not directly reported, it was derived from baseline and follow-up SDs according to the Cochrane Handbook formula: $SD_{change}=\surd (SD_{baseline}^{2}+SD_{\;final}^{2}-2r\;\times \;\;SD_{baseline}\;\times \;\;SD_{\;final})$. A within-group correlation coefficient of r = 0.50 was assumed when not directly estimable. Sensitivity analyses were conducted using alternative plausible values of r = 0.25 and r = 0.75 for the primary LVMI outcome. Accordingly, the random-effects model was prespecified rather than selected solely on the basis of the *I*^2^ statistic (11).

Global inconsistency within the network was assessed using the design-by-treatment interaction model, while local inconsistency was evaluated using the node-splitting method. The relative hierarchy of efficacy for each intervention was determined based on the Surface Under the Cumulative Ranking curve (SUCRA); higher SUCRA values indicate a greater probability of being the most effective treatment (12). Certainty of evidence for the main network estimates was assessed using the CINeMA framework, a structured approach for network meta-analysis aligned with GRADE principles (13, 14). Finally, a comparison-adjusted funnel plot was constructed to detect potential small-study effects or publication bias.

## 3 Results

### 3.1 Characteristics of included studies

**Figure 1:**
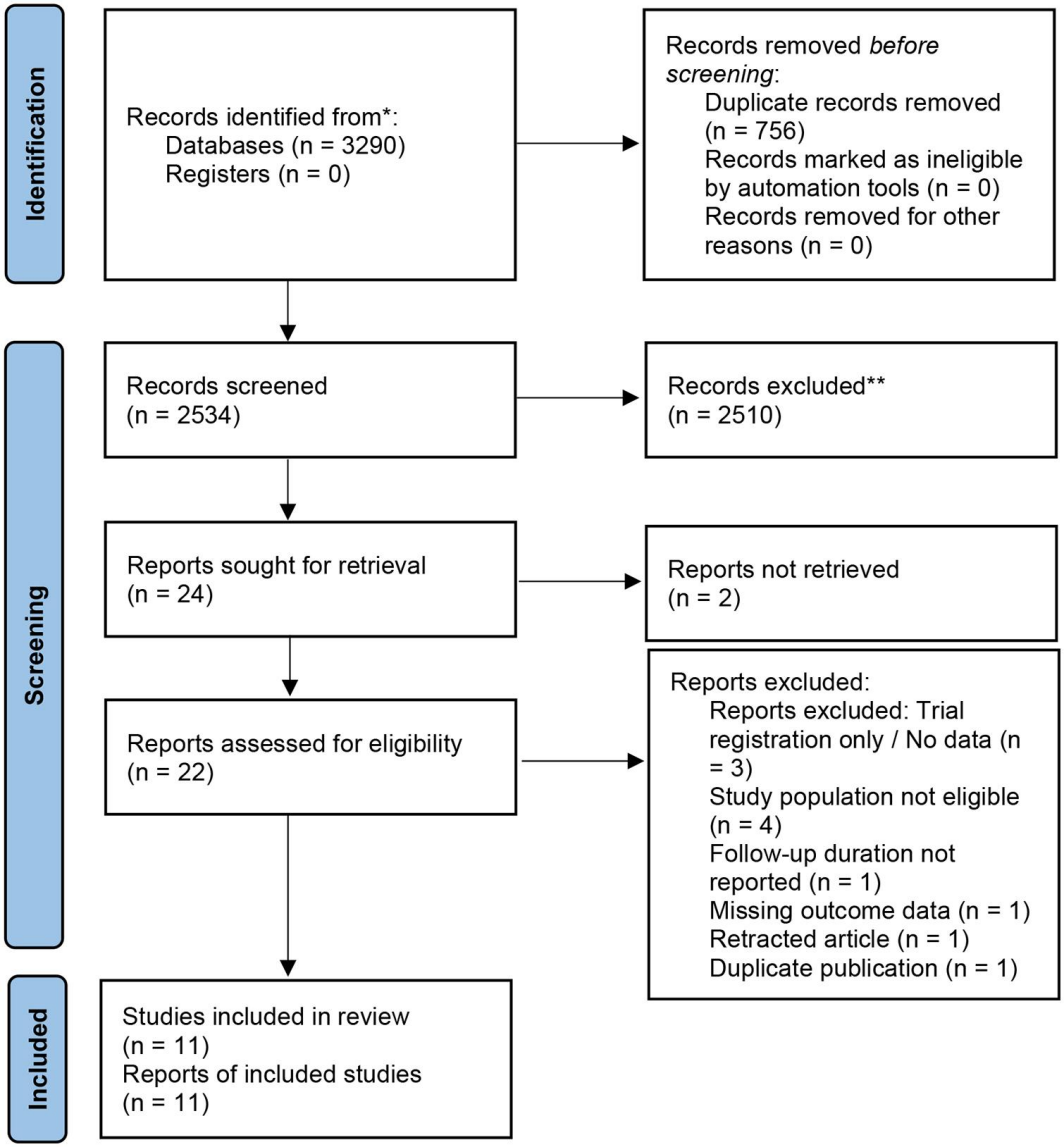
PRISMA flowchart.

**Figure 2:**
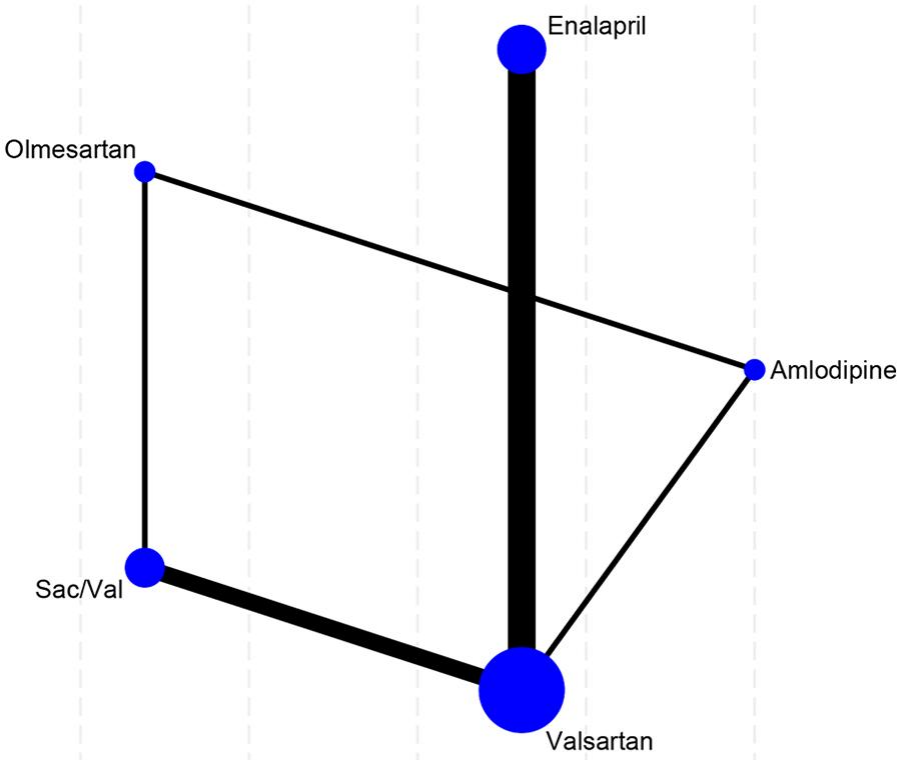
Evidence network of included studies.

Adhering to the PRISMA flowchart (Figure 1), a total of 11 RCTs (8, 15–24) were ultimately included, comprising 851 patients with essential hypertension and evidence of cardiovascular remodeling. We constructed a network of evidence centered around valsartan, Sac/Val, enalapril, olmesartan, and amlodipine (Figure 2). Detailed baseline characteristics of the intervention groups and the specific search strategies are presented in Table 1 and Supplementary Table S2, respectively. The baseline LVMI across studies ranged from 64 to 166 g/m^2^. Imaging assessments included echocardiography (*n* = 9) and CMR (*n* = 2), with some studies including populations with early-stage remodeling characterized by elevated pulse pressure. Potential effect modifiers varied across comparisons, including baseline LVMI, imaging modality, diabetes status, follow-up duration, and phenotype definition. One Sac/Val trial enrolled patients with elevated pulse pressure rather than imaging-confirmed LVH.

**Table 1:** Characteristics of included studies.

Study (year)	Country	Study design	Intervention (daily dose, mg/d)	Sample size (n)	Population	Imaging modality	Age (mean ± SD, y)	Male (%)	Follow-up (weeks)	Baseline LVMI (mean ± SD, g/m^2^)
Lee et al. (8)	Singapore	PROBE	Sac/Val (400) vs. Val (160)	38/39	HTN + LVH	CMR	58 ± 11	41.0	52	64 ± 19 vs. 64 ± 18
An et al. (22)	China	RCT	Sac/Val (200–400) vs. Val (160–320)	50/50	HTN + LVH + T2DM	ECHO	70.2 ± 7.9	85.0	24	128.6 ± 16.5 vs. 130.3 ± 25.4
Zhang et al. (19)	China	RCT	Sac/Val (100–400) vs. Val (80–240)	30/30	HTN + LVH	ECHO	59.6 ± 13.5	63.3	48	130.8 ± 20.3 vs. 123.9 ± 16.8
Li and Zhang (23)	China	RCT	Valsartan (80) vs. Ena (20)	40/30	HTN + LVH	ECHO	56 ± 7	60.0	24	148.8 ± 33.2 vs. 149.7 ± 32.4
Zheng (21)	China	RCT	Val (80–160) vs. Ena (10–30)	40/40	HTN + LVH	ECHO	54 ± 7	67.5	24	148.9 ± 33.1 vs. 149.7 ± 32.4
Yasunari et al. (15)	Japan	Double-blind RCT	Val (80) vs. Aml (5)	50/50	HTN + LVH	ECHO	63.0 ± 11.5	59.6	32	166 ± 39 vs. 161 ± 39
Zhu and Lin (20)	China	RCT	Val (80) vs. Ena (10)	45/41	HTN + LVH	ECHO	NR	64.0	12	146.8 ± 20.5 vs. 146.3 ± 21.3
Yang et al. (24)	China	RCT	Val (80) vs. Ena (10)	26/24	HTN + LVH	ECHO	52.1 ± 9.2	58.0	16	148.7 ± 18.7 vs. 150.2 ± 20.2
Nalbantgil et al. (18)	Turkey	Double-blind RCT	Val (80) vs. Ena (20)	20/20	HTN + LVH	ECHO	54.1 ± 5.3	100.0	24	162.1 ± 22.4 vs. 165.0 ± 24.2
Schmieder et al. (17)	Germany	Double-blind RCT	Sac/Val (200–400) vs. Olm (20–40)	57/57	HTN + elevated PP	CMR	59.8 ± 10.7	67.5	52	72.1 ± 18.0 vs. 72.1 ± 12.0
Rosendorff et al. (16)	USA	Double-blind RCT	Aml (5–10) vs. Olm (20–40)	36/38	HTN + LVH	ECHO	63.9 ± 11.7	99.0	52	122.6 ± 35.4 vs. 116.9 ± 29.6

Data are presented as mean ± SD, number (percentage), or number of participants (n).

RCT, randomized controlled trial; PROBE, prospective randomized open blinded end-point; HTN, hypertension; LVH, left ventricular hypertrophy; T2DM, type 2 diabetes mellitus; PP, pulse pressure; CMR, cardiac magnetic resonance; ECHO, echocardiography; Sac/Val, sacubitril/valsartan; Val, valsartan; Ena, enalapril; Aml, amlodipine; Olm, olmesartan; SD, standard deviation; NR, not reported.

### 3.2 Risk of bias assessment

**Figure 3:**
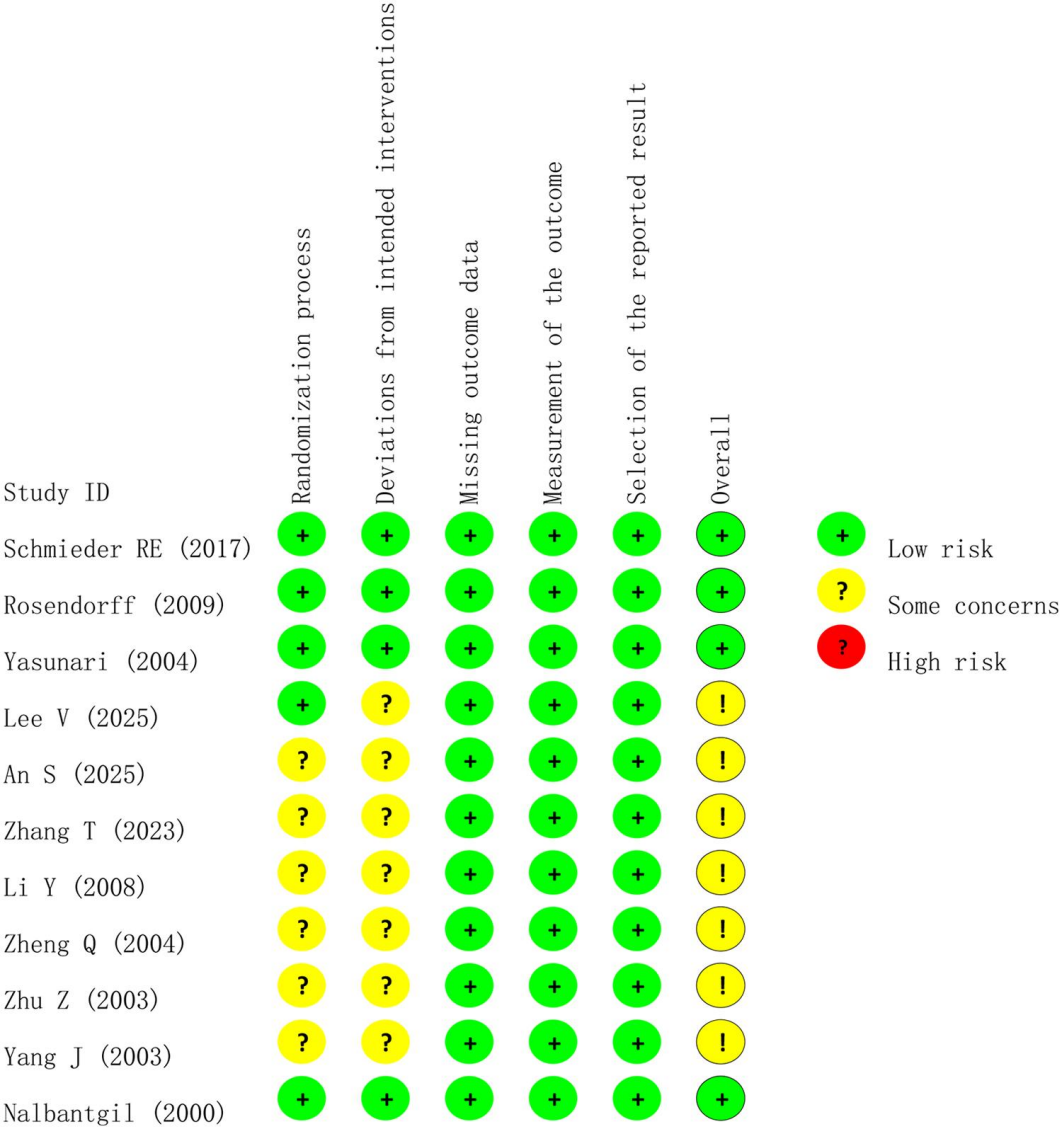
Results of bias risk assessment for included studies.

Quality assessment was performed using the Cochrane RoB 2.0 tool for the 11 included studies (Figure 3), with a summary of bias risk percentages provided in Supplementary Figure S1. Overall, four double-blind RCTs (15–18) were rated as “Low Risk” across all domains, providing high-quality evidence. One study utilizing a PROBE design (8) was flagged with “Some Concerns” due to the unblinded nature of the intervention; however, detection bias was effectively mitigated as LVMI was measured blindly by an independent third-party core laboratory. The remaining six studies (19–24) were rated as “Some Concerns” in the domains of “Randomization Process” or “Deviations from Intended Interventions”, primarily due to insufficient descriptions of allocation concealment or blinding implementation. All included studies demonstrated low risk regarding missing outcome data and measurement of the outcome, indicating good data integrity and reliability of the endpoints.

### 3.3 Relative efficacy on LVMI

#### 3.3.1 Pairwise and network meta-analysis

**Figure 4:**
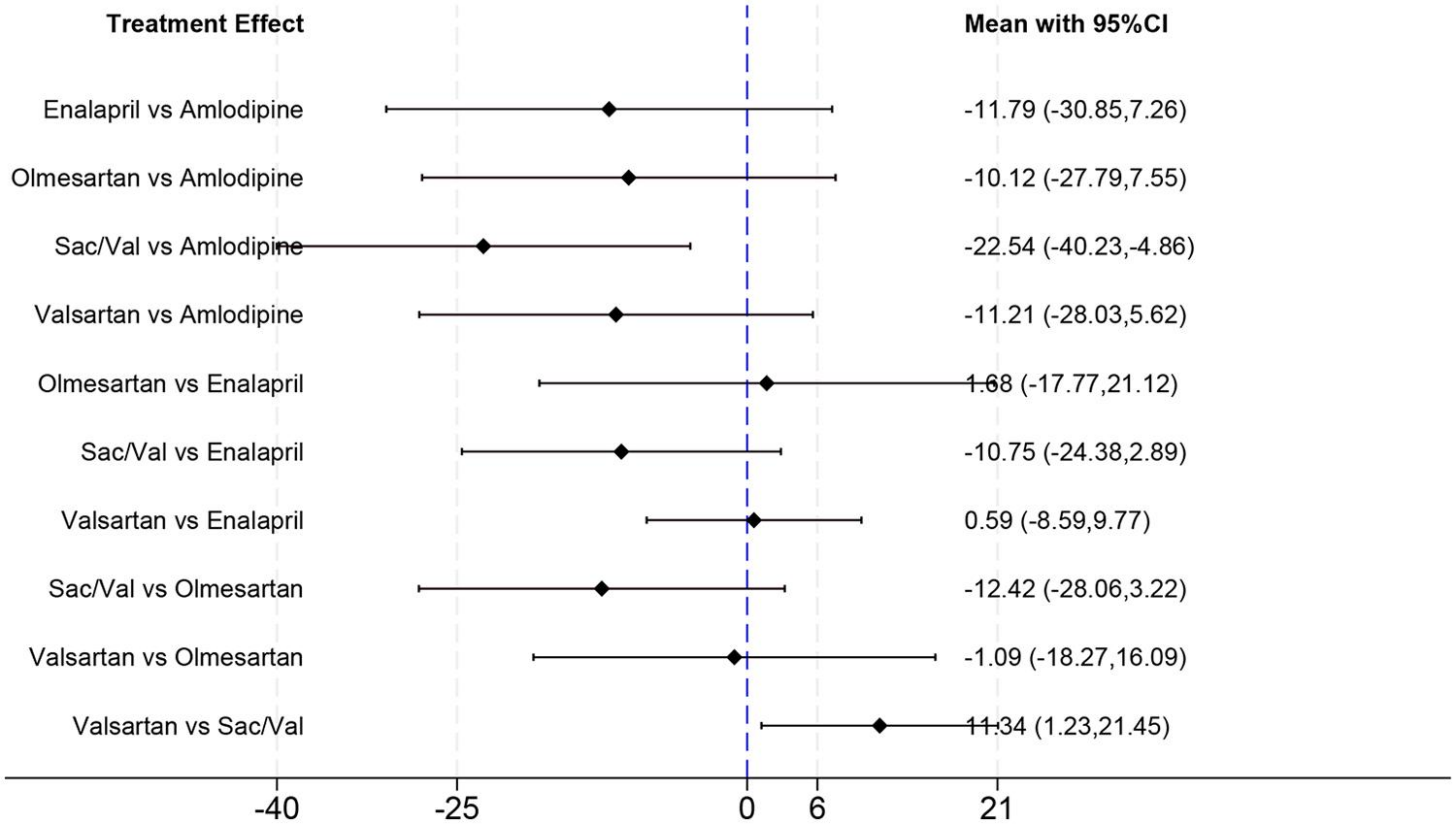
Interval plot of network meta-analysis showing the effects of different interventions on LVMI.

Under the random-effects model, Sac/Val showed the most favorable point estimates for LVMI regression in the network (Table 2, Figure 4). Compared to the standard CCB amlodipine, Sac/Val significantly reduced LVMI (MD = −22.54 g/m^2^; 95% CI: −40.23, −4.86). Compared with valsartan, sacubitril/valsartan was associated with greater LVMI regression (MD = −11.34 g/m^2^; 95% CI: −21.45, −1.23).

**Table 2:** Pairwise comparisons of the efficacy of each intervention in reducing LVMI.

Amlodipine	Valsartan	Sac/val	Olmesartan	Enalapril
**Amlodipine**	−11.21 (−28.03, 5.62)	**−22.54** (−40.23, −4.86)*	−10.12 (−27.79, 7.55)	−11.79 (−30.85, 7.26)
11.21 (−5.62, 28.03)	**Valsartan**	**−11.34** (−21.45, −1.23)*	1.09 (−16.09, 18.27)	−0.59 (−9.77, 8.59)
**22.54** (4.86, 40.23)*	**11.34** (1.23, 21.45)*	**Sac/Val**	12.42 (−3.22, 28.06)	10.75 (−2.89, 24.38)
10.12 (−7.55, 27.79)	−1.09 (−18.27, 16.09)	−12.42 (−28.06, 3.22)	**Olmesartan**	−1.68 (−21.12, 17.77)
11.79 (−7.26, 30.85)	0.59 (−8.59, 9.77)	−10.75 (−24.38, 2.89)	1.68 (−17.77, 21.12)	**Enalapril**

Data are presented as mean difference (MD) and 95% confidence interval (CI). Comparisons should be read as column vs. row. Bold values with an asterisk (*) indicate statistical significance.

Compared with enalapril and olmesartan, Sac/Val also showed numerically greater LVMI regression (MD = −10.75 and −12.42 g/m^2^, respectively), but these differences were not statistically significant. No statistically significant differences were observed among the remaining active comparators.

#### 3.3.2 Probability of efficacy ranking

**Figure 5:**
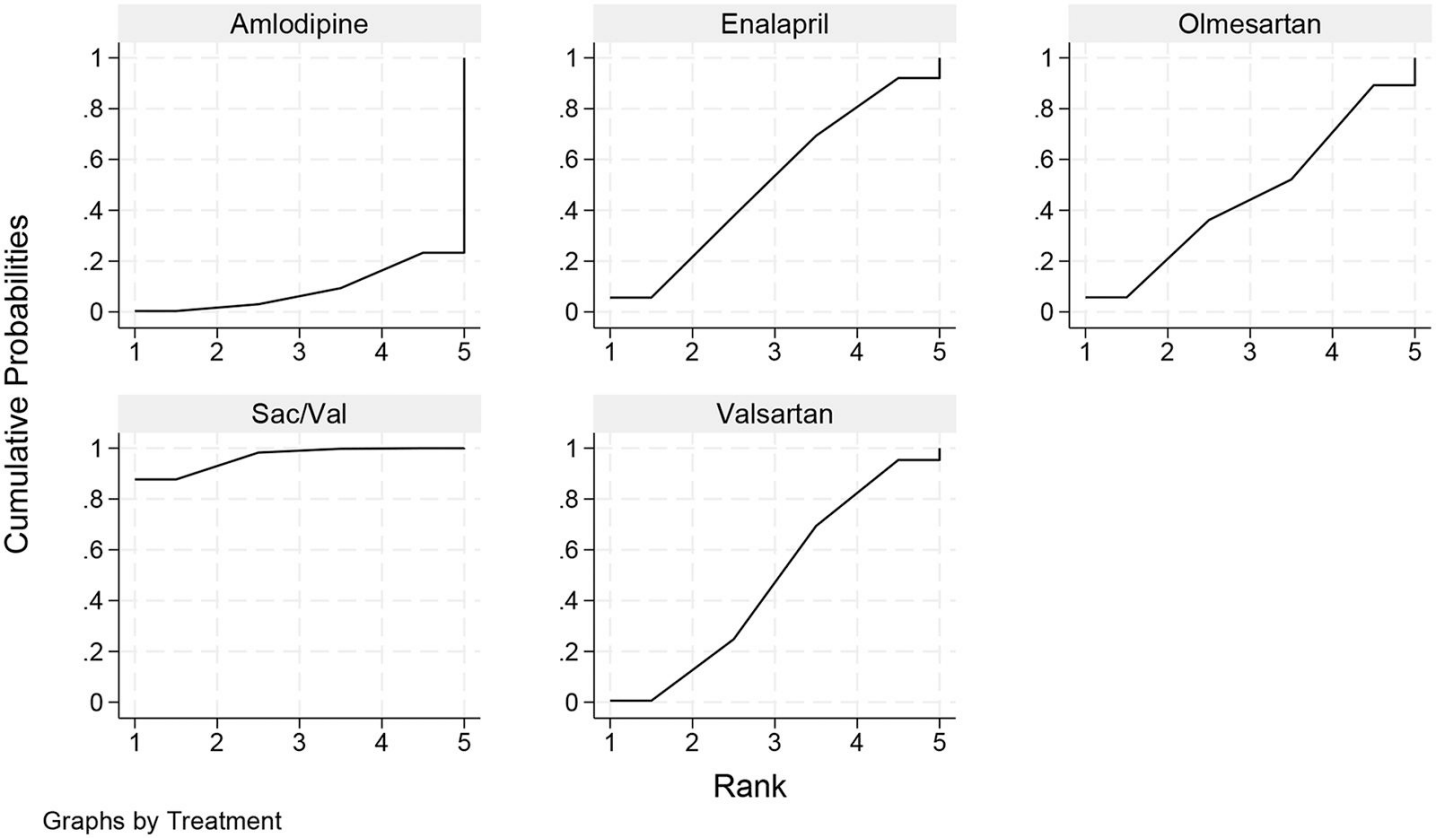
Cumulative ranking probability curves of SUCRA for each intervention measure.

SUCRA cumulative probability analysis provided a supportive ranking summary (Figure 5). Sac/Val had the highest SUCRA value (96.4%), followed by enalapril (51.2%), valsartan (47.5%), olmesartan (45.8%), and amlodipine (9.0%). However, given the sparse network structure, substantial heterogeneity, and observed incoherence, treatment ranking should be interpreted cautiously and should not be considered more informative than the corresponding effect estimates and confidence intervals. Thus, although Sac/Val ranked highest for LVMI regression, comparative interpretation was based primarily on effect sizes and their uncertainty rather than on ranking probabilities alone.

### 3.4 Assessment of inconsistency

Global inconsistency assessed by the design-by-treatment interaction model was statistically significant (*χ*^2^ = 8.08, *P* = 0.0045), indicating disagreement between direct and indirect evidence in the LVMI network. Node-splitting results are presented in Supplementary Table S3 and showed significant local incoherence for amlodipine vs. olmesartan, amlodipine vs. valsartan, olmesartan vs. sacubitril/valsartan, and sacubitril/valsartan vs. valsartan (all *P* = 0.004). No significant local incoherence was observed for enalapril vs. valsartan (*P* = 0.968), although the corresponding indirect estimate was imprecise. The certainty of evidence for the primary LVMI comparisons, assessed using the CINeMA framework, is presented in Supplementary Table S4 and ranged from low to very low, mainly because of imprecision, heterogeneity, and network incoherence. Accordingly, comparative claims were limited to cautious interpretation of direction and uncertainty rather than strong assertions of superiority across the entire network.

### 3.5 Heterogeneity and sensitivity analysis

**Figure 6:**
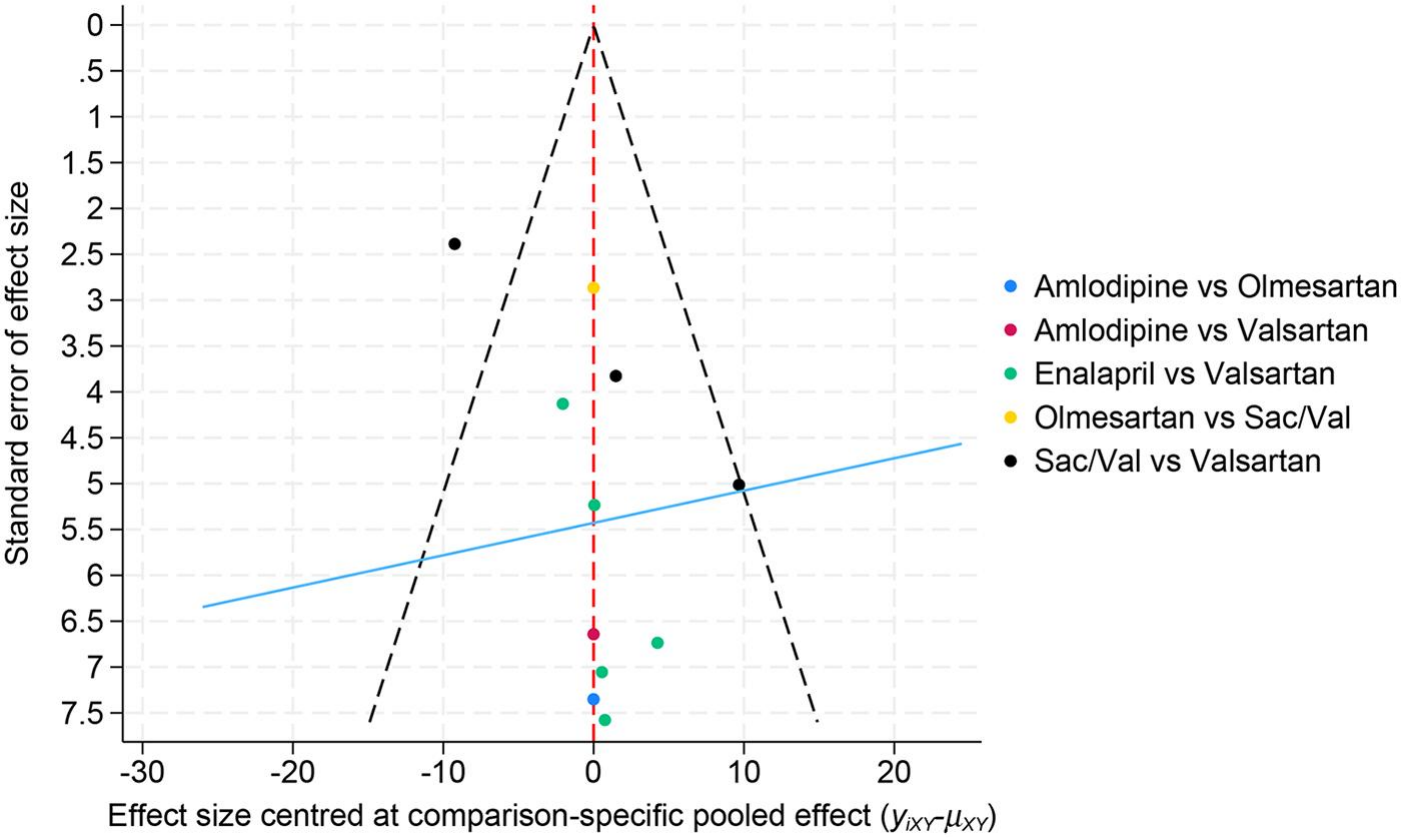
Comparison-corrected funnel plot for assessing publication bias.

Given the high global heterogeneity observed in the network model (*I*^2^ = 74.9%), a leave-one-out sensitivity analysis was conducted to identify influential studies. Zhang et al. (19) was retained in the primary network meta-analysis and was excluded only in sensitivity analysis to assess its influence on heterogeneity and treatment effect estimates. After exclusion of this study, global heterogeneity decreased to 66.0%, while the overall direction of effect remained unchanged (MD = −20.37 g/m^2^), indicating that the main finding was directionally robust despite persisting between-study heterogeneity. Publication bias assessment using the comparison-adjusted funnel plot showed no obvious small-study effects (Figure 6). We further evaluated several planned restriction analyses to assess the robustness of the primary LVMI findings under more homogeneous or methodologically restricted conditions. In the echocardiography-restricted network, the Sac/Val-vs.-valsartan comparison remained estimable and directionally consistent with the primary analysis (MD = −19.31 g/m^2^, 95% CI −25.28 to −13.35). In the imaging-confirmed LVH-restricted network, the Sac/Val-vs.-valsartan comparison also remained estimable and directionally consistent, although the magnitude of effect was attenuated (MD = −14.03 g/m^2^, 95% CI −21.56 to −6.50). However, both restricted subnetworks became tree-shaped and lacked a closed loop, precluding formal incoherence assessment. In the low-risk-of-bias–restricted network, only the Sac/Val-vs.-olmesartan comparison remained available, and the estimate was imprecise and not statistically significant (MD = −3.28 g/m^2^, 95% CI −8.90 to 2.34). Because the Sac/Val-vs.-valsartan comparison was no longer represented and the restricted network had no closed loop, this analysis provided only very limited reassessment of the primary LVMI conclusion. A CMR-restricted analysis was not feasible because only two CMR-based trials were available and they informed different treatment comparisons, preventing a stable connected subnetwork. Sensitivity analyses using alternative assumed within-group correlation coefficients for imputed change-score SDs (r = 0.25 and 0.75) yielded materially similar LVMI network estimates compared with the primary analysis (r = 0.50). Across all assumptions, sacubitril/valsartan remained significantly superior to amlodipine and valsartan, whereas comparisons with enalapril and olmesartan remained non-significant. Treatment ranking was also unchanged, with sacubitril/valsartan remaining the top-ranked intervention (SUCRA 96.0% at r = 0.25, 96.4% at r = 0.50, and 96.7% at r = 0.75). Residual heterogeneity increased at higher r values, but the overall interpretation of the network did not materially change (Supplementary Table S6).

### 3.6 Secondary outcome analysis

We systematically evaluated SBP, DBP, and LVEF as secondary outcomes. Sac/Val ranked highest for both SBP and DBP reduction (SUCRA > 98%). Therefore, greater blood pressure lowering may plausibly explain at least part of the observed LVMI benefit, and the current study cannot distinguish blood-pressure-mediated effects from potential BP-independent effects. No significant between-group differences were observed for LVEF in the available network, suggesting no clear differential effect on systolic function across the included comparisons (relevant interval plots, cumulative ranking curves, and detailed data are shown in Supplementary Figures S3–S5 and Supplementary Tables S7–S9). LVEF data were available only for Sac/Val, valsartan, and enalapril; no eligible amlodipine- or olmesartan-based trials reported extractable LVEF change data. Given the predominantly preserved systolic function of the included populations and the absence of a parallel synthesis of adverse events or discontinuation, LVEF should be regarded as a neutral safety-context outcome rather than a key discriminator of comparative efficacy.

## 4 Discussion

By constructing a network meta-analysis model, this study compared the relative efficacy of Sac/Val and conventional monotherapies for reversing hypertensive cardiovascular remodeling. Pooled data from 11 RCTs suggested that Sac/Val showed the most favorable point estimates within the current evidence network. This finding differs from the pattern reported in early meta-analyses, such as that by Fagard et al., which positioned ARBs as the preferred class for reversing left ventricular hypertrophy (25). It is also broadly consistent with evidence of reverse cardiac remodeling reported in recent heart failure studies (26, 27).

Although Sac/Val was numerically superior to all comparators, the differences compared with enalapril and olmesartan did not reach statistical significance. This phenomenon—where a substantial numerical advantage is accompanied by marginal statistical significance—may be primarily attributed to the limited sample sizes of current studies and the resulting wide confidence intervals. Notably, sensitivity analysis showed that the overall direction of effect remained unchanged after exclusion of the clinically influential Zhang et al. (19) trial, supporting the robustness of the main finding. These findings suggest that sacubitril/valsartan may offer greater LVMI regression than some comparators, although larger head-to-head trials are needed before firm comparative conclusions can be drawn.

Sac/Val also ranked highest for SBP and DBP reduction in the present analysis. A blood-pressure-mediated contribution to the observed LVMI regression is plausible. ARNI-related non-hemodynamic mechanisms have been described in experimental and translational studies (28–30), but the present analysis does not allow separation of BP-mediated and BP-independent effects. Formal meta-regression on achieved blood pressure change was not feasible because achieved BP change was inconsistently reported across studies and the network was too sparse for a statistically reliable analysis. Any mechanistic interpretation beyond blood pressure reduction remains hypothesis-generating.

The analysis of LVEF did not show statistically significant differences between groups; clinically, this should be interpreted as a neutral finding rather than a lack of efficacy. In the available studies, Sac/Val was not associated with an apparent deterioration in pump function. This pattern of structural improvement without an evident between-group difference in LVEF is broadly consistent with prior studies of sacubitril/valsartan (31, 32). These findings suggest that Sac/Val may be used without a clear adverse effect on systolic function in patients with early hypertensive heart disease, although the available LVEF evidence remains limited.

Reversal of LVMI has been associated with a reduced risk of composite cardiovascular endpoints (6, 33). The anti-remodeling signal observed with Sac/Val supports increasing attention to target-organ protection in addition to blood pressure control in hypertension management (3, 4). For patients with persistent LV remodeling despite conventional RAS inhibition, sacubitril/valsartan may be considered a hypothesis-generating alternative for further study; however, the current comparative evidence remains insufficient to support routine preferential switching on the basis of LVMI regression alone.

This study has several limitations. (1) We pooled data from echocardiography and CMR, which may have introduced uncertainty because LV mass assessment is not fully interchangeable across imaging modalities (34). Supplementary Table S1 summarizes key study-level effect modifiers to support a qualitative assessment of transitivity; however, this assumption could not be fully verified because several older trials incompletely reported relevant covariates. In addition, formal stratified analyses by age, follow-up duration, or baseline LVMI were not statistically reliable in this small and sparse network. (2) The limited number of studies for some secondary outcomes and network nodes resulted in wide confidence intervals and reduced statistical precision. (3) Existing evidence is largely based on follow-up periods of less than one year; whether short-term reversal of LVMI translates into long-term clinical benefit requires further confirmation. (4) Both the design-by-treatment interaction model and node-splitting analysis suggested inconsistency within the LVMI network, which further reduces confidence in the pooled estimates and the stability of treatment ranking.

## 5 Conclusion

This network meta-analysis suggests that sacubitril/valsartan may be associated with greater LVMI regression than some conventional antihypertensive comparators; however, the robustness of this apparent advantage is limited by substantial heterogeneity, evidence of global and local inconsistency, and low-to-very-low certainty across the key comparisons. Accordingly, these findings should be viewed as provisional rather than definitive.

## Data Availability

The original contributions presented in the study are included in the article/Supplementary Material, further inquiries can be directed to the corresponding author.
