# Supplementary material for: Reversal of left ventricular hypertrophy with sacubitril/valsartan vs. standard antihypertensive agents in hypertension: a systematic review and network meta-analysis
Source: Front Cardiovasc Med. 2026 Apr 14;13:1794041. doi: 10.3389/fcvm.2026.1794041 (PMC13121351; doi:10.3389/fcvm.2026.1794041)
Supplement: Supplementary file 1 [file Datasheet1.docx]

**Supplementary Material**

**Reversal of Left Ventricular Hypertrophy with Sacubitril/Valsartan versus Standard Antihypertensive Agents in Hypertension: A Systematic Review and Network Meta-Analysis**

Supplementary Table 1. Study-level distribution of potential effect modifiers relevant to the transitivity assumption across studies included in the LVMI network

| **Study** | **Comparison** | **Phenotype definition** | **Imaging modality** | **Baseline LVMI/LVM (g/m²)** | **Mean age (y)** | **Male (%)** | **Diabetes status / prevalence** | **Baseline SBP (mmHg)** | **Baseline DBP (mmHg)** | **Follow-up (weeks)** | **Background therapy** | **Potential transitivity concern** |
| --- | --- | --- | --- | --- | --- | --- | --- | --- | --- | --- | --- | --- |
| Lee V (2025) | Sac/Val vs Valsartan | Hypertension + imaging-confirmed LVH | CMR (LVM index) | 64 ± 19 vs 64 ± 18 | 58 ± 11 | 41 | NR | NR | NR | 52 | 2-week ACEI/ARB washout for prior users; additional non-RAAS antihypertensive agents allowed as needed | CMR-based assessment rather than echocardiography; lower baseline LVM index than most echo-based studies; PROBE design |
| An S (2025) | Sac/Val vs Valsartan | Hypertension + LVH + T2DM | Echocardiography (LVMI) | 128.55 ± 16.47 vs 130.27 ± 25.41 | 70.20 ± 7.90 | 85 | 100% vs 100% (T2DM cohort) | 157.16 ± 12.26 vs 155.06 ± 13.75 | 102.46 ± 10.52 vs 101.96 ± 10.81 | 24 | Both groups received empagliflozin; add-on antihypertensive therapy allowed if BP remained uncontrolled | Older diabetic cohort; may differ in remodeling phenotype and treatment response from non-diabetic LVH populations |
| Zhang T (2023) | Sac/Val vs Valsartan | Hypertension + imaging-confirmed LVH | Echocardiography (LVMI) | 130.77 ± 20.30 vs 123.88 ± 16.76 | 59.61 ± 13.54 | 63.3 | 10.0% vs 10.0% | 160.50 ± 11.11 vs 154.67 ± 13.33 | 89.58 ± 10.93 vs 89.33 ± 17.04 | 48 | Low-salt/low-fat lifestyle intervention; dose uptitration of study drugs; no clear washout reported | Typical LVH phenotype, but longer follow-up than several older Chinese echocardiographic trials |
| Li Y (2008) | Valsartan vs Enalapril | Hypertension + imaging-confirmed LVH | Echocardiography (LVMI) | 148.8 ± 33.2 vs 149.7 ± 32.4 | 56 ± 7 | 60 | NR | NR | NR | 24 | Antihypertensive drugs stopped for >2 weeks before treatment | No major phenotype imbalance identified, but baseline BP and diabetes status were not extractable |
| Zheng Q (2004) | Valsartan vs Enalapril | Hypertension + imaging-confirmed LVH | Echocardiography (LVMI) | 148.9 ± 33.1 vs 149.7 ± 32.4 | 54 ± 7 | 67.5 | NR | NR | NR | 24 | NR | No major phenotype imbalance identified, but baseline BP, diabetes status, and background therapy were not extractable |
| Yasunari K (2004) | Valsartan vs Amlodipine | Hypertension + imaging-confirmed LVH | Echocardiography (LVMI) | 166 ± 39 vs 161 ± 39 | 63.0 ± 11.5 | 59.6 | NR | 152 ± 8 vs 152 ± 6 | 93 ± 5 vs 92 ± 6 | 32 | 4-week placebo run-in; participants were untreated or had discontinued antihypertensive agents; no concomitant medications reported at entry | Higher baseline LVMI than several other studies; Japanese cohort; double-blind design |
| Zhu Z (2003) | Valsartan vs Enalapril | Hypertension + imaging-confirmed LVH | Echocardiography (LVMI) | 146.8 ± 20.5 vs 146.3 ± 21.3 | NR | 64 | NR | 157.75 ± 9.65 vs 159.32 ± 9.36 | 98.35 ± 7.36 vs 98.16 ± 6.89 | 12 | NR | Age not reported; shortest follow-up, which may limit comparability with longer remodeling studies |
| Yang J (2003) | Valsartan vs Enalapril | Hypertension + imaging-confirmed LVH | Echocardiography (LVMI) | 148.7 ± 18.7 vs 150.2 ± 20.2 | 52.1 ± 9.2 | 58 | NR | 159.9 ± 11.1 vs 159.6 ± 19.2 | 94.2 ± 8.9 vs 96.7 ± 5.8 | 16 | Hydrochlorothiazide 25 mg/day could be added if BP response was insufficient after 4 weeks | Short follow-up, which may limit comparability with longer remodeling studies |
| Nalbantgil S (2000) | Valsartan vs Enalapril | Hypertension + imaging-confirmed LVH | Echocardiography (LVMI) | 162.1 ± 22.4 vs 165.0 ± 24.2 | 54.1 ± 5.3 | 100 | NR | 166.4 ± 7.8 vs 164.8 ± 7.7 | 101.1 ± 3.6 vs 100.3 ± 3.3 | 24 | 2-week placebo run-in before randomization | All-male cohort and high baseline LVMI; may not be fully comparable with mixed-sex studies |
| Schmieder RE (2017) | Sac/Val vs Olmesartan | Hypertension + elevated pulse pressure | CMR (LVMI) | 72.1 ± 18.0 vs 72.1 ± 12.0 | 59.8 ± 10.7 | 67.5 | NR | 155.3 ± 9.0 vs 155.0 ± 9.1 | 92.7 ± 8.8 vs 91.7 ± 8.7 | 52 | Protocol-based dose uptitration to BP target; no washout/background cointervention details extracted for this table | Population defined by elevated pulse pressure rather than imaging-confirmed LVH; CMR-based assessment; substantially lower baseline LVMI than echo-based LVH studies |
| Rosendorff C (2009) | Amlodipine vs Olmesartan | Hypertension + imaging-confirmed LVH | Echocardiography (LVMI) | 122.6 ± 35.4 vs 116.9 ± 29.6 | 63.9 ± 11.7 | 99 | 19.2% vs 14.0% | 164.9 ± 13.8 vs 163.2 ± 18.1 | 92.1 ± 13.6 vs 92.2 ± 12.1 | 52 | 1- to 3-week washout/screening period; open-label hydrochlorothiazide and terazosin permitted according to titration algorithm | Near all-male cohort; relatively lower baseline LVMI; background add-on therapy frequently required |

**Abbreviations:** LVMI, left ventricular mass index; LVM, left ventricular mass; LVH, left ventricular hypertrophy; CMR, cardiac magnetic resonance; SBP, systolic blood pressure; DBP, diastolic blood pressure; T2DM, type 2 diabetes mellitus; NR, not reported or not extractable.

**Note:** Potential effect modifiers relevant to transitivity are summarized at the study level across treatment comparisons. Data are presented as reported in the original studies. Diabetes status/prevalence and relevant background treatment features are shown when available. NR indicates not reported or not extractable from the published report.

Supplementary Table 2. Detailed Literature Search Strategy in PubMed

| **Step** | **Search Query** |
| --- | --- |
| **#1** | "Hypertension"[Mesh] OR Hypertension[tiab] OR Hypertens*[tiab] OR "high blood pressure"[tiab] OR "Hypertensive Heart Disease"[tiab] OR HHD[tiab] |
| **#2** | "Hypertrophy, Left Ventricular"[Mesh] OR "Ventricular Remodeling"[Mesh] OR "left ventricular hypertrophy"[tiab] OR LVH[tiab] OR "left ventricular mass"[tiab] OR "LV mass"[tiab] OR LVM[tiab] OR LVMI[tiab] OR "cardiac remodeling"[tiab] OR "ventricular remodeling"[tiab] |
| **#3** | "Angiotensin-Converting Enzyme Inhibitors"[Mesh] OR "Angiotensin Receptor Antagonists"[Mesh] OR "Mineralocorticoid Receptor Antagonists"[Mesh] OR "Neprilysin"[Mesh] OR ACEI[tiab] OR ACE inhibitor*[tiab] OR ARB[tiab] OR Angiotensin Receptor Blocker*[tiab] OR ARNI[tiab] OR Sacubitril[tiab] OR Valsartan[tiab] OR LCZ696[tiab] OR Entresto[tiab] OR Enalapril[tiab] OR Ramipril[tiab] OR Perindopril[tiab] OR Benazepril[tiab] OR Lisinopril[tiab] OR Losartan[tiab] OR Candesartan[tiab] OR Irbesartan[tiab] OR Telmisartan[tiab] OR Olmesartan[tiab] OR Spironolactone[tiab] OR Eplerenone[tiab] OR Finerenone[tiab] |
| **#4** | "Randomized Controlled Trial"[Publication Type] OR randomized[tiab] OR placebo[tiab] OR "clinical trial"[tiab] |
| **#5** | **#1 AND #2 AND #3 AND #4** |

Supplementary Table 3. Node-splitting assessment of local incoherence for evaluable comparisons in the LVMI network

| **Comparison** | **Direct estimate** | **Direct SE** | **Indirect estimate** | **Indirect SE** | **Difference** | **Difference SE** | ***P* value** |
| --- | --- | --- | --- | --- | --- | --- | --- |
| Amlodipine vs Olmesartan | 4.10 | 9.08 | -36.67 | 11.11 | 40.77 | 14.35 | 0.004 |
| Amlodipine vs Valsartan | -26.00 | 8.51 | 14.82 | 11.54 | -40.82 | 14.34 | 0.004 |
| Enalapril vs Valsartan | 0.68 | 4.74 | -24.50 | 633.88 | 25.18 | 633.89 | 0.968 |
| Olmesartan vs Sac/Val | -3.28 | 6.05 | -44.07 | 13.02 | 40.79 | 14.35 | 0.004 |
| Sac/Val vs Valsartan | 14.03 | 3.84 | -26.80 | 13.83 | 40.84 | 14.36 | 0.004 |

Note: Node-splitting was performed for evaluable comparisons in the LVMI network. Significant disagreement between direct and indirect evidence was considered suggestive of local inconsistency.

Supplementary Table 4. CINeMA-based certainty assessment for the primary LVMI comparisons

| **Comparison** | **Within-study bias** | **Reporting bias** | **Indirectness** | **Imprecision** | **Heterogeneity** | **Incoherence** | **Overall confidence** |
| --- | --- | --- | --- | --- | --- | --- | --- |
| Sac/Val vs Amlodipine | Some concerns | Some concerns | Some concerns | Some concerns | Major concerns | Major concerns | Low |
| Sac/Val vs Valsartan | Some concerns | Some concerns | Some concerns | Some concerns | Major concerns | Major concerns | Very low |
| Sac/Val vs Enalapril | Some concerns | Some concerns | Some concerns | Major concerns | Major concerns | Some concerns | Very low |
| Sac/Val vs Olmesartan | Some concerns | Some concerns | Some concerns | Major concerns | Major concerns | Major concerns | Very low |

**Abbreviations:** CINeMA, Confidence in Network Meta-Analysis.
**Note:** Judgments were based on RoB 2 assessments, the comparison-adjusted funnel plot, variability in imaging modality and phenotype definition across studies, width of confidence intervals, residual heterogeneity, and inconsistency diagnostics from the design-by-treatment interaction model and node-splitting analysis.

Supplementary Table 5. Structural interpretability of planned restriction analyses and implications for the primary LVMI conclusion

| **Planned restriction analysis** | **Operational definition** | **Studies retained (n)** | **Nodes retained (n)** | **Connected network?** | **Direct comparisons retained** | **Key structural issue after restriction** | **Feasibility for formal NMA interpretation** | **Primary Sac/Val-related comparison(s) retained** | **Can the primary LVMI conclusion be directly reassessed?** | **Direction of effect vs primary analysis** | **Result summary / interpretive implication** |
| --- | --- | --- | --- | --- | --- | --- | --- | --- | --- | --- | --- |
| Echocardiography-restricted network | Include studies using echocardiographic LVMI only; exclude CMR-based studies | 9 | 5 | Yes | Amlodipine–Olmesartan (1); Amlodipine–Valsartan (1); Enalapril–Valsartan (5); Sac/Val–Valsartan (2) | Removal of both CMR studies eliminates the Sac/Val–olmesartan edge and leaves a tree-shaped network without a closed loop; olmesartan is informed only through a single amlodipine–olmesartan study | Limited; analyzable as an exploratory connected network, but incoherence cannot be meaningfully assessed and several estimates depend on single-study bridges | Sac/Val–Valsartan | Partially | Unchanged | Sac/Val versus valsartan remained estimable and directionally consistent with the primary analysis (MD -19.31 g/m², 95% CI -25.28 to -13.35), but the tree-shaped network without a closed loop limited full-network interpretation. |
| Imaging-confirmed LVH-restricted network | Include only studies enrolling patients with imaging-confirmed LVH; exclude the elevated pulse pressure study | 10 | 5 | Yes | Amlodipine–Olmesartan (1); Amlodipine–Valsartan (1); Enalapril–Valsartan (5); Sac/Val–Valsartan (3) | Excluding the elevated pulse pressure trial removes the only direct Sac/Val–olmesartan comparison and again yields a tree-shaped network without a closed loop | Limited; potentially reportable as exploratory only, with weakened transitivity concern but reduced network structure | Sac/Val–Valsartan | Partially | Unchanged, attenuated | Sac/Val versus valsartan remained estimable and directionally consistent, although the effect size was attenuated (MD -14.03 g/m², 95% CI -21.56 to -6.50); the loop-free network precluded formal incoherence assessment. |
| Low-risk-of-bias-restricted network | Restrict to studies judged low risk across RoB 2 domains (Yasunari 2004, Rosendorff 2009, Schmieder 2017, Nalbantgil 2000) | 4 | 5 | Yes | Amlodipine–Olmesartan (1); Amlodipine–Valsartan (1); Enalapril–Valsartan (1); Olmesartan–Sac/Val (1) | Only four studies remain, each contributing a unique edge; there are no replicated direct comparisons, no closed loop, and no heterogeneity degrees of freedom | Very limited; only fixed-effect exploratory analysis was feasible, and formal NMA interpretation remained unstable despite nominal connectivity | Sac/Val–Olmesartan only | Very limited | Not directly comparable | Only the Sac/Val-versus-olmesartan comparison remained available and was imprecise (MD -3.28 g/m², 95% CI -8.90 to 2.34), so the primary LVMI conclusion could not be robustly reassessed. |

Supplementary Table 6. Sensitivity analyses using alternative assumed within-group correlation coefficients for imputed LVMI change-score SDs

| **Assumed r** | **Sac/Val vs Amlodipine, MD (95% CI)** | **Sac/Val vs Valsartan, MD (95% CI)** | **Sac/Val vs Enalapril, MD (95% CI)** | **Sac/Val vs Olmesartan, MD (95% CI)** | **SUCRA (Sac/Val)** | **tau²** | **I²** |
| --- | --- | --- | --- | --- | --- | --- | --- |
| 0.25 | -21.85 (-39.71, -3.99) | -11.26 (-20.86, -1.66) | -10.76 (-24.12, 2.59) | -10.71 (-25.77, 4.36) | 96.0 | 62.01 | 65.33% |
| 0.50 | -22.54 (-40.23, -4.86) | -11.34 (-21.45, -1.23) | -10.75 (-24.38, 2.89) | -12.42 (-28.06, 3.22) | 96.4 | 75.02 | 74.87% |
| 0.75 | -23.20 (-40.79, -5.62) | -11.34 (-21.94, -0.75) | -10.66 (-24.57, 3.25) | -14.24 (-30.49, 2.00) | 96.7 | 88.81 | 85.41% |

**Note:** Change-score SDs were imputed from baseline and final SDs using assumed within-group correlation coefficients of r = 0.25, 0.50, and 0.75. Across all assumptions, the direction, statistical significance pattern, and treatment ranking of the primary LVMI network remained materially similar, although residual heterogeneity increased at higher r values.

Supplementary Table 7. Pairwise comparisons of the efficacy of each intervention in reducing systolic blood pressure

| **Amlodipine** | **Valsartan** | **Sac/Val** | **Olmesartan** | **Enalapril** |
| --- | --- | --- | --- | --- |
| **Amlodipine** | 1.60 (-2.93, 6.12) | **-7.49 (-13.05, -1.94)*** | -0.22 (-6.73, 6.30) | 2.28 (-3.39, 7.96) |
| -1.60 (-6.12, 2.93) | **Valsartan** | **-9.09 (-13.09, -5.09)*** | -1.81 (-7.32, 3.70) | 0.69 (-2.65, 4.03) |
| **7.49 (1.94, 13.05)*** | **9.09 (5.09, 13.09)*** | **Sac/Val** | **7.28 (2.54, 12.01)*** | **9.77 (4.56, 14.99)*** |
| 0.22 (-6.30, 6.73) | 1.81 (-3.70, 7.32) | **-7.28 (-12.01, -2.54)*** | **Olmesartan** | 2.50 (-3.92, 8.92) |
| -2.28 (-7.96, 3.39) | -0.69 (-4.03, 2.65) | **-9.77 (-14.99, -4.56)*** | -2.50 (-8.92, 3.92) | **Enalapril** |

Note: Data are presented as mean difference (MD) and 95% confidence interval (CI). Comparisons should be read as column vs row. Bold values with an asterisk (*) indicate statistical significance. Negative MD values favor the column-defining treatment for blood pressure reduction.

Supplementary Table 8. Pairwise comparisons of the efficacy of each intervention in reducing diastolic blood pressure

| **Amlodipine** | **Valsartan** | **Sac/Val** | **Olmesartan** | **Enalapril** |
| --- | --- | --- | --- | --- |
| **Amlodipine** | 1.53 (-0.45, 3.51) | **-5.15 (-8.16, -2.14)*** | -2.11 (-5.59, 1.37) | 1.80 (-0.64, 4.24) |
| -1.53 (-3.51, 0.45) | **Valsartan** | **-6.68 (-9.23, -4.14)*** | -3.64 (-7.00, -0.27) | 0.27 (-1.16, 1.69) |
| **5.15 (2.14, 8.16)*** | **6.68 (4.14, 9.23)*** | **Sac/Val** | **3.05 (0.21, 5.88)*** | **6.95 (4.03, 9.87)*** |
| 2.11 (-1.37, 5.59) | 3.64 (0.27, 7.00) | **-3.05 (-5.88, -0.21)*** | **Olmesartan** | 3.90 (0.25, 7.56) |
| -1.80 (-4.24, 0.64) | -0.27 (-1.69, 1.16) | **-6.95 (-9.87, -4.03)*** | -3.90 (-7.56, -0.25) | **Enalapril** |

Note: Data are presented as mean difference (MD) and 95% confidence interval (CI). Comparisons should be read as column vs row. Bold values with an asterisk (*) indicate statistical significance. Negative MD values favor the column-defining treatment for blood pressure reduction.

Supplementary Table 9. Pairwise comparisons of the effects of each intervention on left ventricular ejection fraction

| **Comparator 1** | **Comparator 2** | **MD (95% CI)** |
| --- | --- | --- |
| **Enalapril** | Valsartan | 1.31 (-9.32, 6.70) |
| **Sac/Val** | Valsartan | 0.49 (-7.99, 7.00) |
| **Enalapril** | Sac/Val | 0.82 (-2.02, 3.66) |

Note: only trials with extractable LVEF change data were included in this analysis; amlodipine and olmesartan were not represented because no eligible studies provided usable LVEF change data.


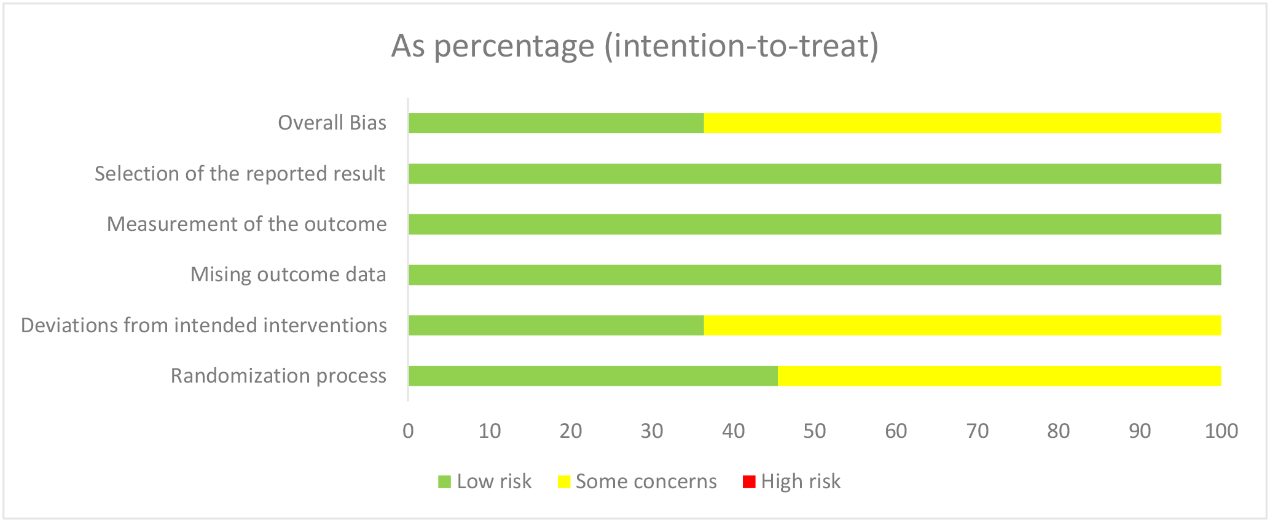


Supplementary Figure 1. Summary Chart of Bias Risk Percentage


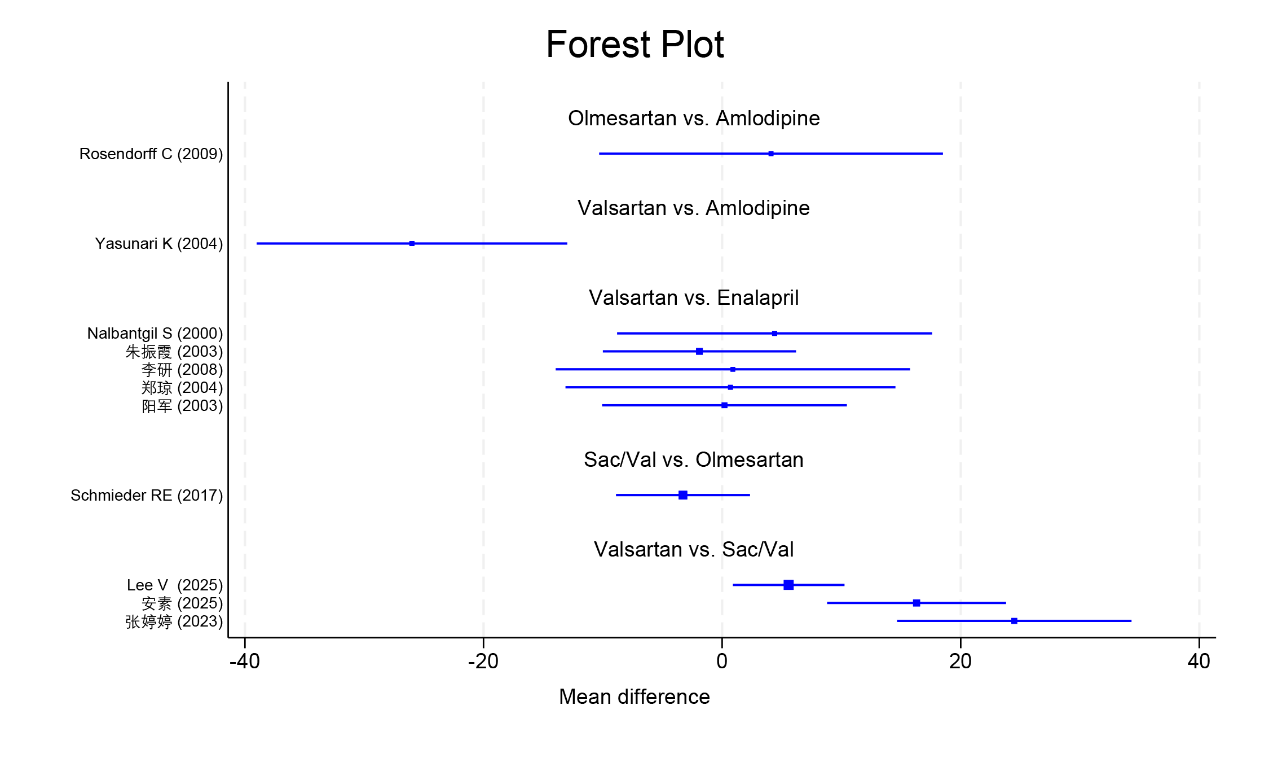


Supplementary Figure 2. Forest plot for direct comparison of left ventricular mass index


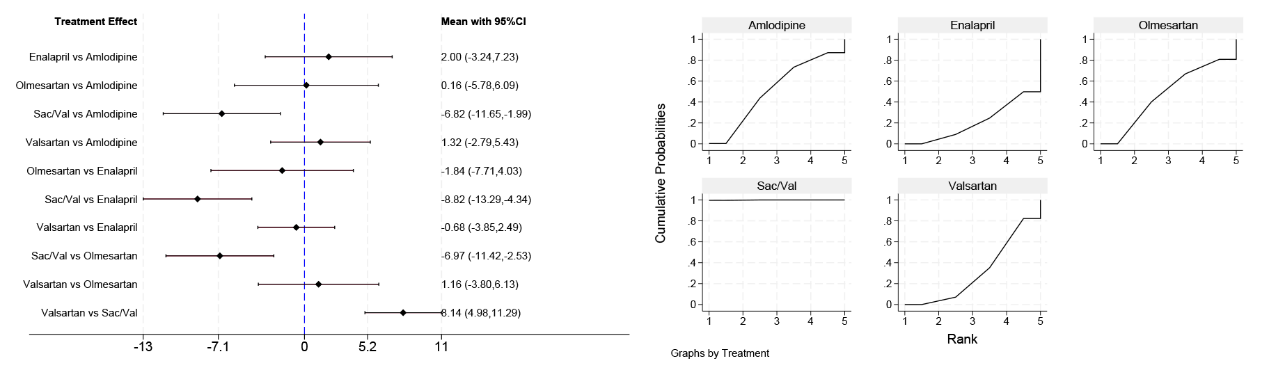


Supplementary Figure 3. Network Meta-analysis Results of Systolic Blood Pressure


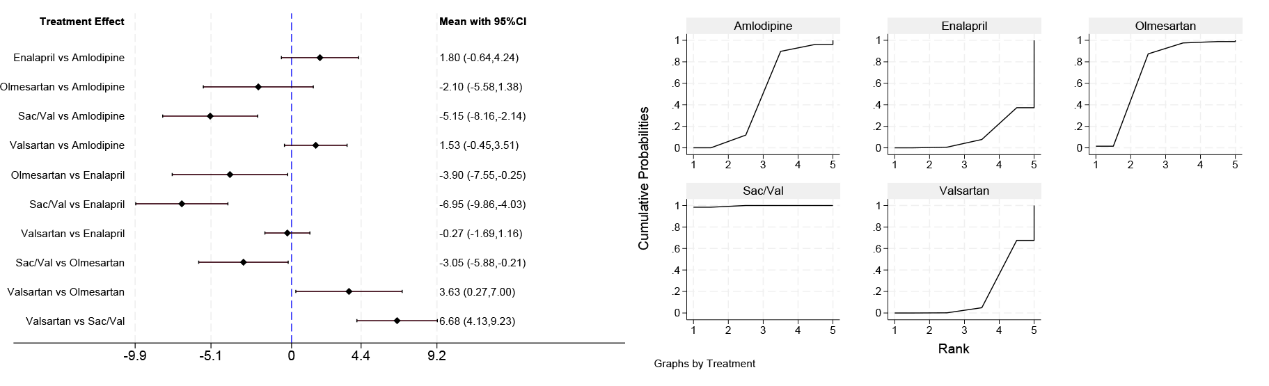


Supplementary Figure 4. Network Meta-analysis Results of Diastolic Blood Pressure


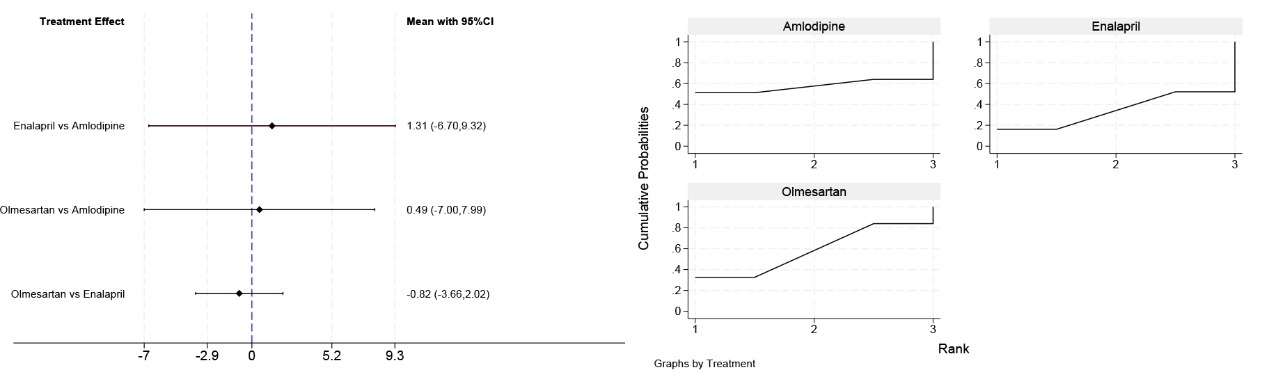


Supplementary Figure 5. Results of the network meta-analysis of left ventricular ejection fraction
